# Supplementary material for: Pervasive duplication of tumor suppressors in Afrotherians during the evolution of large bodies and reduced cancer risk
Source: eLife. 2021 Jan 29;10:e65041. doi: 10.7554/eLife.65041 (PMC7952090; doi:10.7554/eLife.65041)
Supplement: Supplementary file 1. [file elife-65041-supp1.docx]

**Supplementary File 1. Summary of duplications in Atlantogenata**

| **Species** | **Common Name** | **Number of** | | **Percent of Genes** | | **Mean ECNC/Hit** |
| --- | --- | --- | --- | --- | --- | --- |
|  |  | **Hits** | **Duplicated** | **Found** | **Duplicated** |  |
| *Choloepus hoffmanni* | Hoffmans Two-Toed Sloth | 14082 | 3204 | 78.19% | 22.75% | 0.98 |
| *Chrysochloris asiatica* | Cape Golden Mole | 13547 | 2716 | 75.22% | 20.05% | 0.99 |
| *Dasypus novemcinctus* | Nine-Banded Armadillo | 13819 | 2605 | 76.73% | 18.85% | 0.98 |
| *Echinops telfairi* | Lesser Hedgehog Tenrec | 12903 | 1670 | 71.64% | 12.94% | 0.99 |
| *Elephantulus edwardii* | Cape Elephant Shrew | 12884 | 3048 | 71.53% | 23.66% | 0.99 |
| *Elephas maximus* | Asian Elephant | 14073 | 907 | 78.14% | 6.44% | 1 |
| *Loxodonta africana* | African Savanna Elephant | 14051 | 940 | 78.01% | 6.69% | 1 |
| *Loxodonta cyclotis* | African Forest Elephant | 14065 | 900 | 78.09% | 6.40% | 1 |
| *Mammut americanum* | American Mastodon | 13840 | 737 | 76.84% | 5.33% | 1 |
| *Mammuthus columbi* | Columbian Mammoth | 13059 | 426 | 72.51% | 3.26% | 1 |
| *Mammuthus primigenius* | Woolly Mammoth | 13935 | 723 | 77.37% | 5.19% | 1 |
| *Orycteropus afer* | Aardvark | 13880 | 1083 | 77.06% | 7.80% | 0.99 |
| *Palaeoloxodon antiquus* | Straight Tusked Elephant | 13969 | 745 | 77.56% | 5.33% | 1 |
| *Procavia capensis* | Rock Hyrax | 13672 | 788 | 75.91% | 5.76% | 1 |
| *Trichechus manatus* | Manatee | 14092 | 1046 | 78.24% | 7.42% | 1 |
