# Supplementary material for: Pervasive duplication of tumor suppressors in Afrotherians during the evolution of large bodies and reduced cancer risk
Source: eLife. 2021 Jan 29;10:e65041. doi: 10.7554/eLife.65041 (PMC7952090; doi:10.7554/eLife.65041)
Supplement: Supplementary file 2. [file elife-65041-supp2.docx]

**Supplementary File 2. RNA-Seq datasets used in this study, along with key biological and genome information.**

| **Organism** | **Common Name** | **Genome** | **SRA Acc.** | **Tissues** |
| --- | --- | --- | --- | --- |
| *Dasypus novemcinctus* | Nine-banded armadillo | dasNov3 | SRR494779, SRR494767, SRR494780, SRR494770, SRR309130, SRR494771, SRR4043756, SRR494776, SRR494778, SRR4043762, SRR4043755, SRR6206923, SRR4043761, SRR4043760, SRR6206913, SRR4043763, SRR494772, SRR494781, SRR494774, SRR494777, SRR494775, SRR4043754, SRR1289524, SRR4043758, SRR6206903, SRR1289523, SRR4043759, SRR3222425, SRR494768, SRR494769, SRR6206908, SRR4043757, SRR494766, SRR6206918, SRR494773 | Kidney, Spleen, Cerebellum W/ Brainstem, Rt. Quadricep, Mid-Stage Pregnant Endometrium, Cervix, Lung, Liver, Skeletal Muscle, Ascending Colon, Pregnant Armadillo Endometrium, Heart, Placenta |
| *Loxodonta africana* | African savanna elephant | loxAfr3, loxAfrC, loxAfr4 | SRR6307198, SRR1041765, SRR6307199, SRR6307201, SRR6307196, SRR6307202, SRR6307200, SRR6307195, SRR975188, SRR6307194, SRR6307204, SRR3222430, SRR6307205, SRR975189, SRR6307197, SRR6307203 | Blood, Fibroblast, Placenta |
| *Trichechus manatus latirostris* | Manatee | triMan1, triManLat2 | SRR4228542, SRR4228545, SRR4228544, SRR4228539, SRR4228541, SRR4228538, SRR4228546, SRR4228537, SRR4228540, SRR4228543, SRR4228547 | Buffy Coat |
