# Supplementary material for: Pervasive duplication of tumor suppressors in Afrotherians during the evolution of large bodies and reduced cancer risk
Source: eLife. 2021 Jan 29;10:e65041. doi: 10.7554/eLife.65041 (PMC7952090; doi:10.7554/eLife.65041)
Supplement: Supplementary file 3. [file elife-65041-supp3.docx]

**Supplementary File 3. Summary of PGLS model used to estimate lifespan.**

| **PGLS: ln(Lifespan) ~ ln(Size)** | |
| --- | --- |
|  | **ln(Lifespan)** |
| **ln(Size)** | 0.200^***^ |
|  | -0.027 |
| **Constant** | 1.327^***^ |
|  | -0.237 |
|  | |
| **Observations** | 28 |
| **Log Likelihood** | -22.16 |
| **Akaike Inf. Crit.** | 50.32 |
| **Bayesian Inf. Crit.** | 54.095 |
|  | |
| *Note:* | ^*^p^**^p^***^p<0.01 |
